# Mechanistic Empirical Design for Circular Concrete Performance with Glass Powder

**DOI:** 10.3390/ma19214740

**Published:** 2026-11-08

**Authors:** Habib Loubani, Madumita Sadagopan, Agnes Nagy

**Affiliations:** Department of Resource Recovery and Building Technology, The Swedish Centre for Resource Recovery (SCRR), Faculty of Textiles, Engineering and Business, University of Borås, SE-501 90 Borås, Sweden; madumita.sadagopan@hb.se (M.S.); agnes.nagy@hb.se (A.N.)

**Keywords:** supplementary cementitious materials, glass powder, circular concrete, k-value, sustainable construction materials, recycled aggregates

## Abstract

This study evaluates finely milled float glass powder (GP) as a supplementary cementitious material (SCM) through a mechanistic–empirical framework based on strength and reactivity assessment. A mechanistic approach was developed to quantify the SCM efficiency of glass powder. Compared with conventional standardized testing procedures, the proposed method enables rapid mechanical performance evaluation with minimal testing. The mechanistic model is evaluated on cementitious systems with GP inclusion and consists of the following stages: (1) Best replacement percentage, as determined by the strength activity index (SAI), evaluated on mortar specimens for 20, 25 and 30% replacements. The highest SAI of 85% is observed for 20% replacement. (2) k-value determination by Abram’s law, wherein compressive strength is used as a reference and GP-based concrete (20% replacement) at 0.42, 0.46, 0.5 and 0.61 water–binder ratios are investigated. This results in an average k-value of 0.5. (3) SAI-based k-value (k_SAI_), which derives the compressive strength of glass at different water–binder ratios from reference concrete strengths by multiplying by the SAI value 85% and determining the k-value (k_SAI_) from the resulting compressive strength values using Abram’s law, resulting in 0.43. (4) Equivalent performance of concrete for frost resistance exposure classes (XF1, XF3); according to the SIS-CEN/TR 16639 Dutch method, wherein, if the SCM concrete shows comparable strength to the reference concrete, testing for exposure classes XF1 and XF3 is not required. GP-based concrete with a k-value of 0.5 surpasses the compressive strength reference values of 45 MPa and 43 MPa, respectively. (5) Recycled aggregate-incorporated concrete with a k-value of 0.5 has also been evaluated for equivalent performance, surpassing that of crushed aggregate reference concrete by 7%, which also makes it frost-resistant for exposure classes XF1 and XF3. This approach has the potential to serve as a practical screening tool for the preliminary evaluation of SCMs, thereby accelerating the development and implementation of sustainable cementitious materials.

## 1. Background

The cement industry, responsible for around 7–8% of total CO_2_ emissions worldwide, is one of the largest industrial contributors. These emissions are primarily attributed to the calcination of limestone during clinker production and the high energy demand required to bring about calcination [1]. Adopting a substitution strategy, where part of the cement is replaced with supplementary cementitious materials (SCMs), can significantly reduce CO_2_ emissions [2].

The concrete industry is working actively towards climate-neutral concrete using SCMs in concrete. When finely milled (mechanical activation), glass waste, with its high SiO_2_ content, displays increased reactivity of its amorphous silica, allowing it to react with calcium hydroxide (CH) to produce calcium silicate hydrates (C-S-H) (chemical contribution), which is referred to as pozzolanic reactivity [3,4,5,6].

Waste glass from demolished windows contains a high proportion of amorphous SiO_2_, which can be activated through fine grinding. Mechanical activation increases the reactivity of amorphous silica, enabling it to react with calcium hydroxide (CH) to form calcium silicate hydrates (C-S-H), a process referred to as pozzolanic activity [3,4,5,6], with an optimum glass replacement level of approximately 20 to 30% [3,7,8]. To enhance the pozzolanic activity, the milling process was optimized using a ball mill to produce glass powder with a particle size distribution comparable to that of cement [9].

This study is part of the Swedish project RE:glass, which aims to recycle float glass from windows from demolished windows. The findings of this project include the following: (1) The development of a milling process to produce glass powder with particle distribution close to cement [9]. (2) An LCA study on the climate reduction potential of GP concrete, which showed that 20% GP replacement of Industri cement (CEM II-A/LL) resulted in a 20% reduction in CO_2_ emissions (cradle–grave) [10].

ASTM C1866 [11] is currently the only standard specifically addressing the use of ground glass powder as a supplementary cementitious material (SCM). The standard recognizes the pozzolanic and cementitious contribution of glass powder through performance requirements, including a minimum 28-day strength activity index of 85%, and 7 days of 80%, rather than through an efficiency coefficient. In contrast, other SCMs, such as fly ash and ground granulated blast-furnace slag (GGBS), are assigned k-values within the framework of the Swedish application standard, allowing their contribution to cement replacement to be considered in concrete mix design. To date, an equivalent k-value concept has not been established for glass powder.

This study investigates a mechanistic testing framework for quantifying the cementitious contribution of glass powder to the compressive strength of mortar and concrete.

### 1.1. Durability of Glass as SCM

Glass types vary in their chemical composition, ranging from alkali-rich to alkali-free and aluminum-rich types. Float glass, commonly found in demolition waste, has a high sodium content of 13% [3]. Laboratory glass such as borosilicate glass contains a low sodium content of approximately 4% [12,13], whereas aluminosilicate glass is alkali-free and has high aluminum content of 10–25% [14]. Differences in alkali content influence the properties of concrete when glass is used as an SCM. The main durability concern is the risk of an alkali–silica reaction (ASR) that causes harmful expansion in concrete.

To limit the alkali content in concrete, different replacement levels of glass powder have been investigated, and the resulting ASR expansions have been noted. Several studies have shown that the lowest expansion is observed at a replacement level of 20%. Another key factor influencing ASR risk is the particle size of glass. Glass powder with a particle size below 75 microns shows the lowest risk of ASR expansion. As shown in Table 1, by limiting the particle size to 53 microns and the replacement levels to 20% [15,16,17], it is possible to use glass powder with a high alkali content. This study investigates glass containing Na_2_O 13.2% and K_2_O 0.4% (composition presented in Table 2); the particle size is reduced to less than 53 microns to mitigate ASR risk, and the particle size distribution is provided in Section 2.

### 1.2. Mechanistic Performance of SCMs

The contribution of SCMs to concrete strength and durability is expressed through an equivalence factor, also known as the k-value, which indicates the efficiency of an SCM in comparison to cement. The mechanistic performance of SCMs can be evaluated using the equivalence factor, expressed as the k-value, which provides a quantitative measure of the effectiveness of an SCM relative to Portland cement, particularly with respect to its contribution to strength development [19].

The effectiveness of an SCM (k-value), can be assessed using different methods, such as the strength-based approach, where compressive strength is measured for concrete containing SCMs at different water–binder ratios. This approach focuses on the contribution of SCMs to the mechanical properties of concrete, typically measured through compressive strength development in comparison to reference concrete, where Abram’s law is used to establish an efficiency factor for a specific SCM. This method has been used in the following research articles [19,20]. A k-value of 1 indicates performance equivalent to cement, whereas values below 1 indicate lower efficiency. Thus, higher k-values represent a greater contribution of the SCM to strength development and cement replacement.

Previous research using Abram’s law has investigated four different water-to-binder ratios, which results in four different k-values. However, only a single k-value has been reported; as a result [19,20], the approach to deriving a unified k-value is missing. This study demonstrates the unification of different k-values obtained at different water-to-binder ratios, through sensitivity analysis.

The strength activity index (SAI) is a method for evaluating the chemical reactivity of SCM relative to cement by comparing compressive strength, according to ASTM C1866. One way to relate the k-value to the strength activity index (SAI) is that both describe the contribution of an SCM to strength but from different perspectives. The k-value is a design parameter used to represent the cement equivalent efficiency of an SCM across varying water–binder ratios. In contrast, SAI evaluates the relative strength performance of an SCM at a constant water–binder ratio (0.5), making it a useful indicator of pozzolanic activity. SAI provides a standardized comparison of reactivity under fixed conditions, while the k-value supports mix design by adjusting the effective binder content and, consequently, the effective water-to-binder ratio.

**Figure 1:**
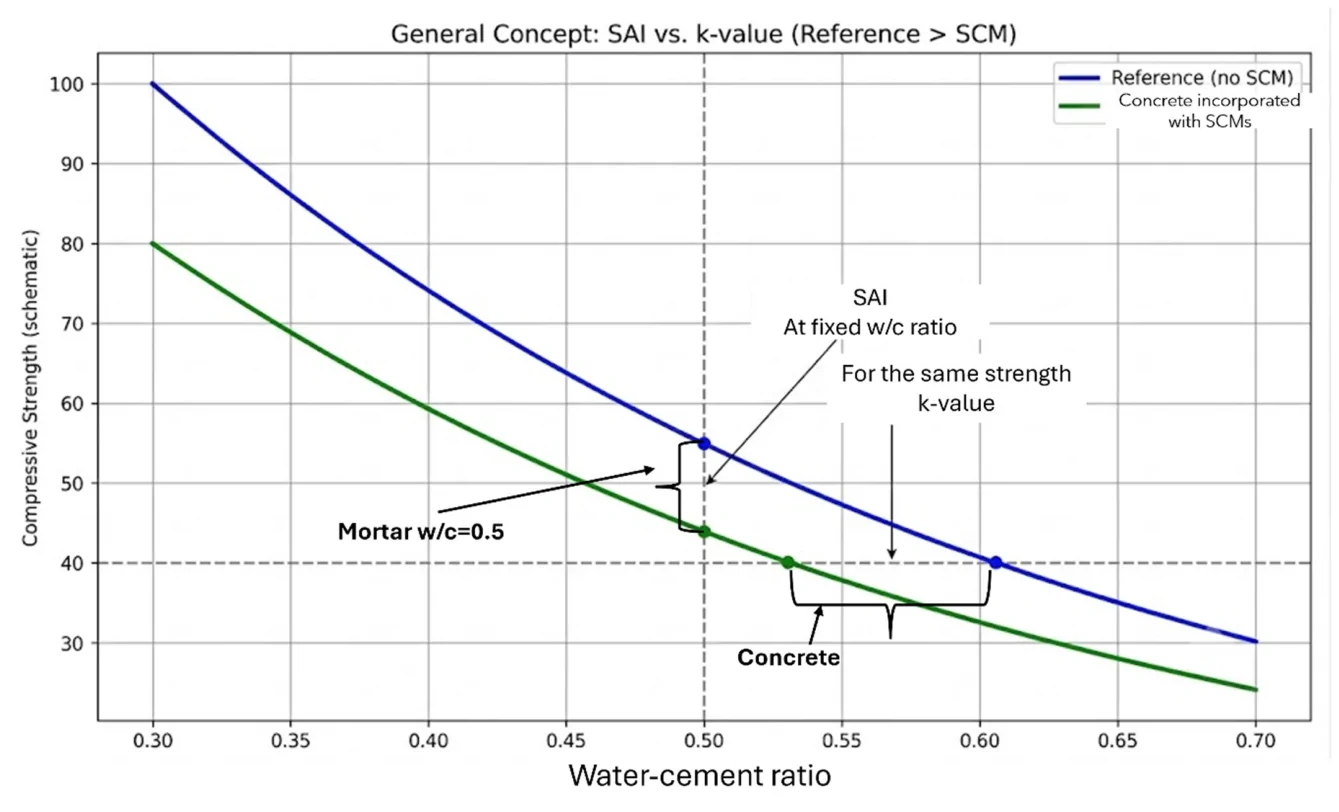
General concept: SAI vs. k-value, where SAI is evaluated on mortar and k-value is evaluated on concrete, generated by AI.

The k-value and SAI are illustrated for concrete and mortar of different water–cement ratios for two types of cementitious systems, a reference cement system, represented by the blue curve, and an SCM-incorporated system, represented by the green curve. For the SAI, the compressive strengths of mixes with different cementitious systems are compared at the same water–cement ratio, which is shown as the vertical difference along the strength axis in Figure 1. For the k-value, the water–binder ratio of the mix with SCM is optimized to give the same strength value as the reference mix. This is shown as the horizontal difference along the water–cement ratio axis in Figure 1. In general, most SCMs achieve lower strength than cement, which corresponds to a lower k-value and SAI, with exceptions such as silica fume.

According to CEN/TR 16639, considering use of the k-value, equivalent concrete performance and equivalent performance of combination concept, the application of the k-value concept varies between member states, reflecting different national practices. Within this context, this report indicates that nine countries use concrete strength, while one country uses mortar compressive strength to assess the efficiency of SCMs [21]. In this study, concrete strength results are used to determine the k-value, while mortar strength results are used to evaluate SAI.

This study evaluated the potential of glass powder as an SCM at a 20% cement replacement level. A mechanistic approach, based on Abram’s law and SAI measurements, is used to quantify the strength contribution of glass powder. This study focuses on float glass, a glass type with a relatively high alkali content (around 13% Na_2_O). Furthermore, the k-value of the glass powder is determined and applied within the equivalent concrete performance concept (ECPC) framework to assess strength and durability in concrete containing natural and recycled aggregates, namely crushed concrete aggregates (CCAs).

The mechanistic approach proposed in this study provides an efficient and scientifically based method for evaluating glass powder as an SCM. Unlike conventional standardized testing procedures, which often require extensive experimental programs, massive quantities of materials, and long testing periods, the proposed method enables rapid evaluation with minimal material consumption. The significance of this approach lies in its ability to quantify the performance of SCM performance in a time- and resource-efficient manner while maintaining a mechanistic basis for evaluation. Furthermore, the method is not limited to glass powder and has the potential to be applied to a wide range of SCMs, providing a practical screening tool for the preliminary assessment of new cementitious materials. Consequently, this approach can accelerate the development and adoption of alternative SCMs while reducing the testing effort, cost, material consumption, and evaluation time.

**Figure 2:**
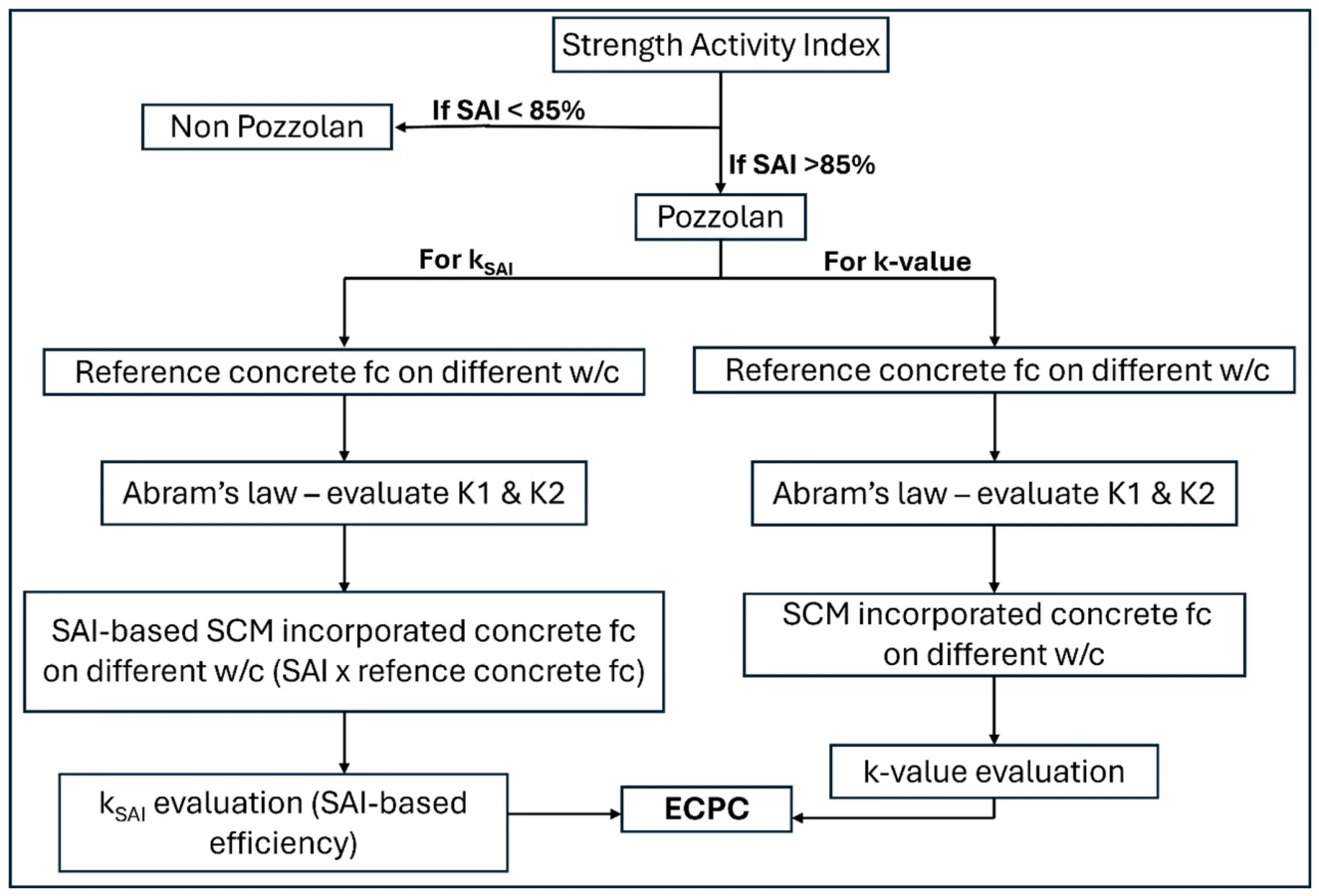
Procedure for mechanistic evaluation of efficiency of SCMs.

The mechanistic procedure for evaluating the efficiency factors of an SCM is outlined in Figure 2. The process begins with the determination of SAI, which is used to assess the pozzolanic behavior of an SCM. Materials with SAI below 85% are classified as non-pozzolanic, whereas materials with SAI above 85% are considered pozzolanic and are subjected to further evaluation, starting with evaluating the best replacement percentage. For both k-value and k_SAI_ approaches, the reference concrete strengths at different water–cement ratios are first determined and used to establish Abram’s law, from which the coefficients K1 and K2 are obtained. For k-value evaluation, the compressive strength (fc) of SCM-incorporated concrete is experimentally determined and used to calculate the efficiency factor. For k_SAI_ evaluation, the compressive strength of the SCM concrete is estimated by multiplying the SAI by the corresponding reference concrete strength. Finally, both approaches can be used within the ECPC framework to evaluate SCM efficiency.

## 2. Materials and Methods

**Figure 3:**
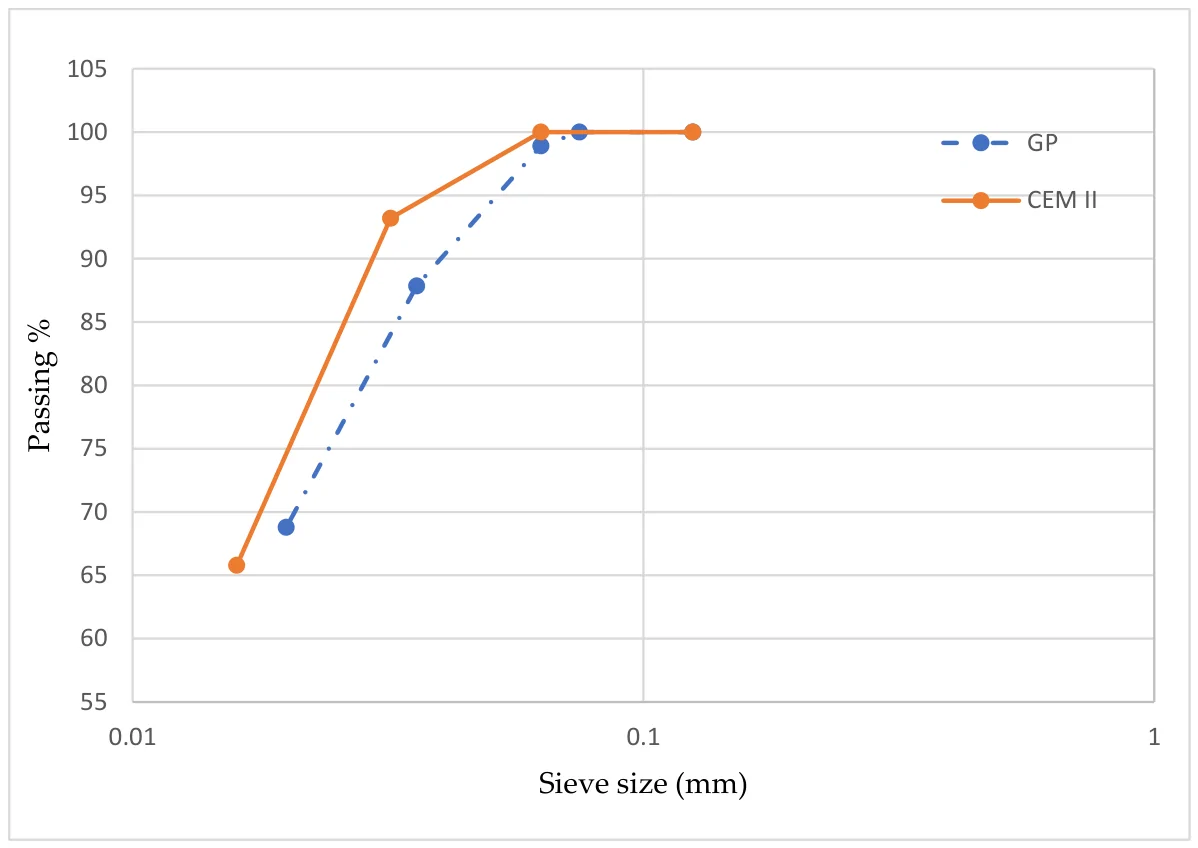
Particle size distribution of glass powder and industrial cement CEM II.

An industrial Portland cement, CEM II/A-LL 52.5R, is used in combination with finely milled glass powder (GP). Cement is partially replaced with 20% GP derived from float glass, which has a density of 2500 kg/m^3^ and is finely milled, with 69% of particles passing 20 microns, using a laboratory-scale ball mill. The preparation involved initial crushing using a jaw crusher (BICO CHIPMUNK CRUSHER WD-64, Burbank, CA, USA) to sizes less than 60 mm, followed by a two-step milling procedure: a rod mill as primary stage to produce coarse powder, and ball mill as the secondary stage to further reduce particle size and enhance reactivity [10]. The particle size distribution and the oxide composition of both CEM II and GP are presented in Figure 3 and Table 2, respectively.

According to the American standard ASTM C1866, glass powder should have at least 90% of its particles finer than 45 microns to ensure sufficient pozzolanic activity [11]. A blended binder system consisting of CEM II/A-LL and 20% glass powder results in a reduction in clinker content comparable to that of pozzolanic (CEM IV) or composite (CEM V) cements, which have clinker contents of 45–89% and 20–64%, respectively. However, such a blend does not constitute a standardized cement under SS-EN 197-1 [22].

Tests for k-value determination

Concrete mixes are prepared using CEM II and GP at four different water–binder ratios (w/b) of 0.61, 0.5, 0.46, and 0.42, hereafter referred to as 60, 50, 46 and 42, respectively, in addition to the k-value and k_SAI_ concrete recipes, which are referred to as kGP and k_SAI_GP, respectively. All mixes are designed with a constant binder content of 370 kg/m^3^. A superplasticizer, Sika Visco-Crete 6720, is incorporated to achieve a workability class of S4 in accordance with SS-EN 206 [23]. The mix proportions are presented in Table 3. The GP mixtures consist of 80% CEM II and 20% GP (GP mixes), while the reference mixtures are prepared with 100% CEM II (CEM II mixes). The aggregate fractions are 0–8 mm natural sand and 8–16 mm crushed stone in the reference concrete mix.

In another theoretical approach, the SAI-based k-value efficiency factor (k_SAI_) is developed. The conventional k-value reflects the overall contribution of GP to concrete performance. Similarly, k_SAI_, derived from SAI, is assumed to provide an efficiency factor comparable to the k-value but with less laboratory investigation. This method follows a procedure similar to that of the conventional k-value determination method but uses SAI as the basis. The compressive strength of the reference concrete is multiplied by the SAI to estimate the corresponding strength of the binder system, which is then used to determine the effective water–cement ratio (w/ceff). Subsequently, Equation (3) is applied to calculate k_SAI_. In simple terms, this method eliminates the need for the time-consuming testing of concrete containing SCMs.

Three cylindrical specimens (100 × 200 mm) are prepared for each concrete mix. After demolding, the specimens are cured in water tanks at 22 °C according to SS-EN 13670 [24].

**Figure 4:**
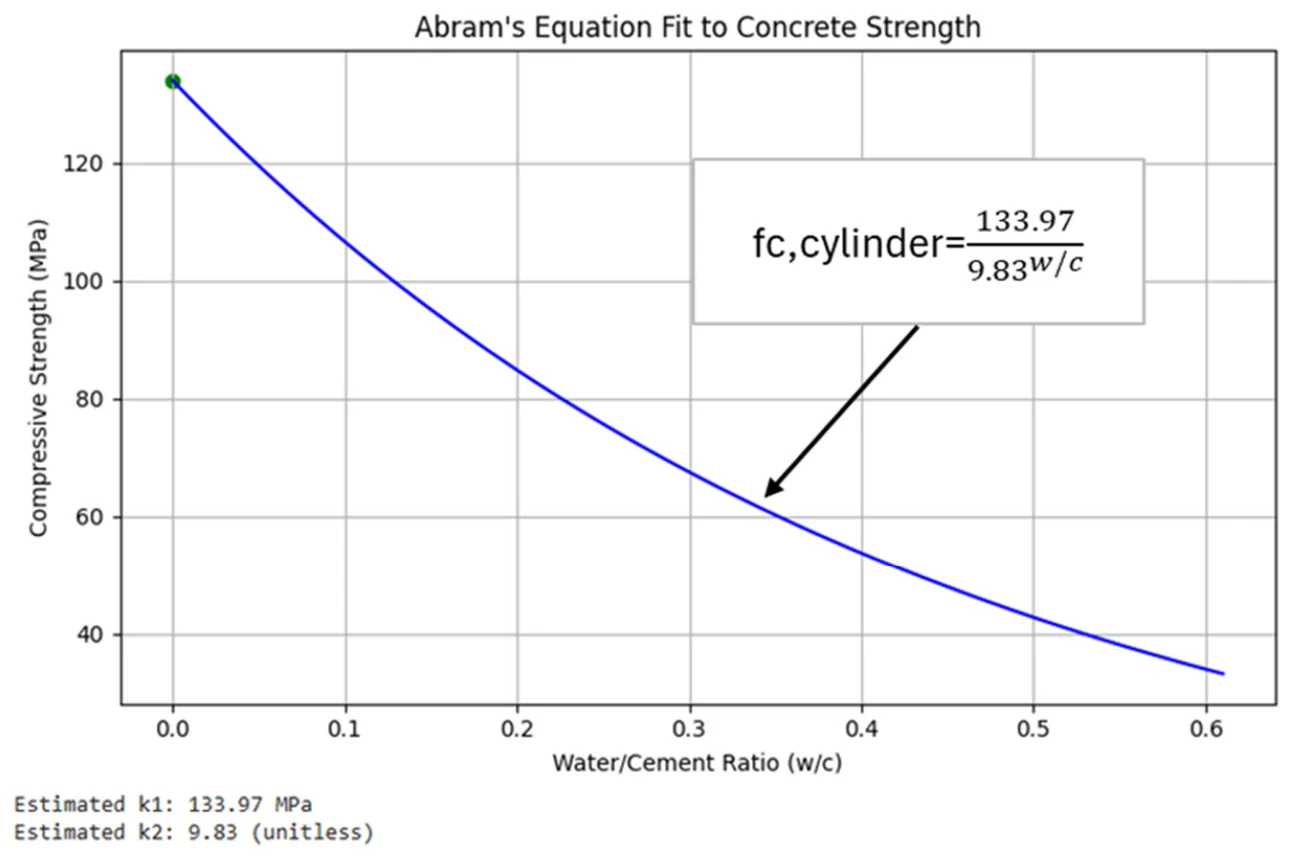
Reference concrete strength curve with K1 and K2 values fitted according to Abram’s law, based on computation in Python, Python code provided in Supplementary Materials, Python code 2.

Abram’s law, given in Equation (1), describes the empirical relationship between the compressive strength and the water–cement ratio (w/c) of reference concrete [19,21,25].

(1) $$f_{c,\mathrm{cylinder}}=\frac{K1}{\;{K2}^{\frac{w}{c}}}$$

where f_c,cylinder_ refers to the compressive strength of concrete, K1 is the intercept factor, K2 is the slope factor, and w/c is the water–cement ratio. Figure 4 illustrates the relationship between compressive strength and the w/c of reference concrete, from which the parameters K1 = 133.9 MPa and K2 = 9.8 were determined. The compressive strengths used to evaluate K1 and K2 are provided in Section 3.2.

The effect of glass powder on the effective cement content, often referred to as the equivalent cement content, can be evaluated using Equation (2). Based on this, the k-value can be determined using Equation (3) [19,25].

(2) $$Ceqv=\frac{W}{w/ceff}$$

where Ceqv refers to the amount of binder equivalent to cement upon using glass powder as an SCM in kg; W refers to the water content in liters; and w/ceff refers to the effective water–cement ratio.

(3) $$k=\frac{Ceqv-c}{A}$$

where k refers to k-value, W is the water content in the concrete mix in liters, w/ceff is the effective water–cement ratio, c refers to the amount of cement in the concrete mix in kg, and A is the amount of glass in the concrete mix in kg.

Tests for SAI determination

Mortar cubes (40 × 40 mm) are also cast in accordance with ASTM C1866 [11] and cured in water at 22 °C in a water bath. The mix proportions used for investigating the SAI for different replacement percentages are presented in Table 4. These cubes are tested for compression to determine the SAI, which is used to evaluate the pozzolanic activity of GP. SAI is the ratio of the mean compressive strength of mortar specimens containing GP to that of CEM II, as shown in Equation (4). According to ASTM C1866 [11], an SCM is considered pozzolanic if it achieves an SAI of at least 75% at 7 days and 85% at 28 days [11].

(4) $$SAI=\frac{CGP}{CEM\;II}$$

where CEM II refers to the compressive strength of mortar cubes containing 100% CEMII, and CGP refers to compressive strength of mortar cubes containing 80% CEM II and 20% GP.

For standardized SCMs, prescribed requirements such as clinker content and maximum water–binder ratio are typically defined. In contrast, this study adopts an approach for non-standardized SCMs, such as GP in Europe, by determining the effective k-value based on strength performance.

As the glass powder is a non-standardized SCM in Europe, its effectiveness is evaluated by comparing the properties of GP concrete properties with those of conventional cement concrete. This follows the equivalent concrete performance concept (ECPC) framework, which is intended for the use of non-standardized SCMs in concrete. Accordingly, concrete containing SCMs is required to demonstrate performance comparable to that of reference concrete in terms of strength and durability, in accordance with the relevant exposure class [21,23,26,27].

The Swedish technical report SIS-CEN/TR 16639:2014 [21] defines the application of ECPC and the requirements for demonstrating equivalent concrete performance. This study considers a typical Swedish outdoor concrete with frost resistance, corresponding to exposure class XF3, concrete exposed to moderate-to-high water saturation without deicing salt.

Further investigations included the use of crushed concrete aggregates (CCAs), which are recycled aggregates produced by crushing concrete waste for reuse in new concrete. Coarse CCA (8/16 mm) was tested for ASR and frost resistance in accordance with RILEM AAR-2 and SS-EN 15304 [28]. In addition, concrete with 100% replacement of coarse natural aggregates by CCA was evaluated for frost resistance without de-icing agents according to SS-EN 15304 [29].

## 3. Results and Discussion

### 3.1. Strength Activity Index

SAI is used to identify the best replacement level among three different levels used in this study for maximizing concrete strength, as evaluated by the 7-day SAI. Three replacement levels of 20%, 25%, and 30% are evaluated, resulting in SAI values of 86, 83, and 82%, respectively, shown in Table 5. Although all tested mixtures satisfy the pozzolanic criteria, since ASTM C1866 specifies that a material should exhibit a minimum of 80% SAI, the 20% replacement level demonstrated the highest SAI and is, therefore, selected as the best replacement level.

Table 6 presents the SAI values of mortar cubes tested at 28 days of curing, which are found to be approximately 85%. According to ASTM C1866, a material exhibiting an SAI of at least 85% at 28 days can be classified as a pozzolan. Therefore, the CGP with the given particle size distribution used in this study can be considered a pozzolanic material and may be used as a potential partial replacement for cement.

### 3.2. Concrete Properties

Concrete mixtures consisting of 100% CEM II Industri Cement and those incorporating a 20% GP replacement are evaluated for compressive strength (fc). Table 7 presents the average compressive strength obtained from three specimens at varying water–cement ratios, along with their slump values indicating workability. Refer to Table 3 for mix proportions.

Concrete containing 20% GP consistently exhibited lower 28-day compressive strength than the reference concrete across all water–binder ratios. The lower strength of GP concrete compared to the reference indicates that the GP is not as effective as cement. By proportioning GP in concrete such that its effect is equivalent to that of cement, i.e., using the efficiency factor (k-value), it may be possible to increase the compressive strength of concrete. Therefore, concrete mixtures were prepared using the k-value determined by Abram’s law.

The workability of concrete containing 20% GP increased compared to the reference mix. This increase in workability can be attributed to the particle size distribution of GP, which enhances packing by working as a bridge between cement and sand particles, thereby improving particle size distribution continuity, in addition to its pozzolanic activity, which contributes to the strength development of GP as an SCM.

It was observed that as the w/b decreased, the strength contribution of glass powder increased. The GP/CEMII strength ratio, which represents the relative contribution of GP to compressive strength compared with cement, increased from 85% at w/b = 0.61 to 95% at w/b = 0.46, before decreasing slightly to 89% at w/b = 0.42. This suggests that GP does not significantly increase water demand.

### 3.3. Compressive Strength Related to k-Value by Abram’s Law

**Figure 5:**
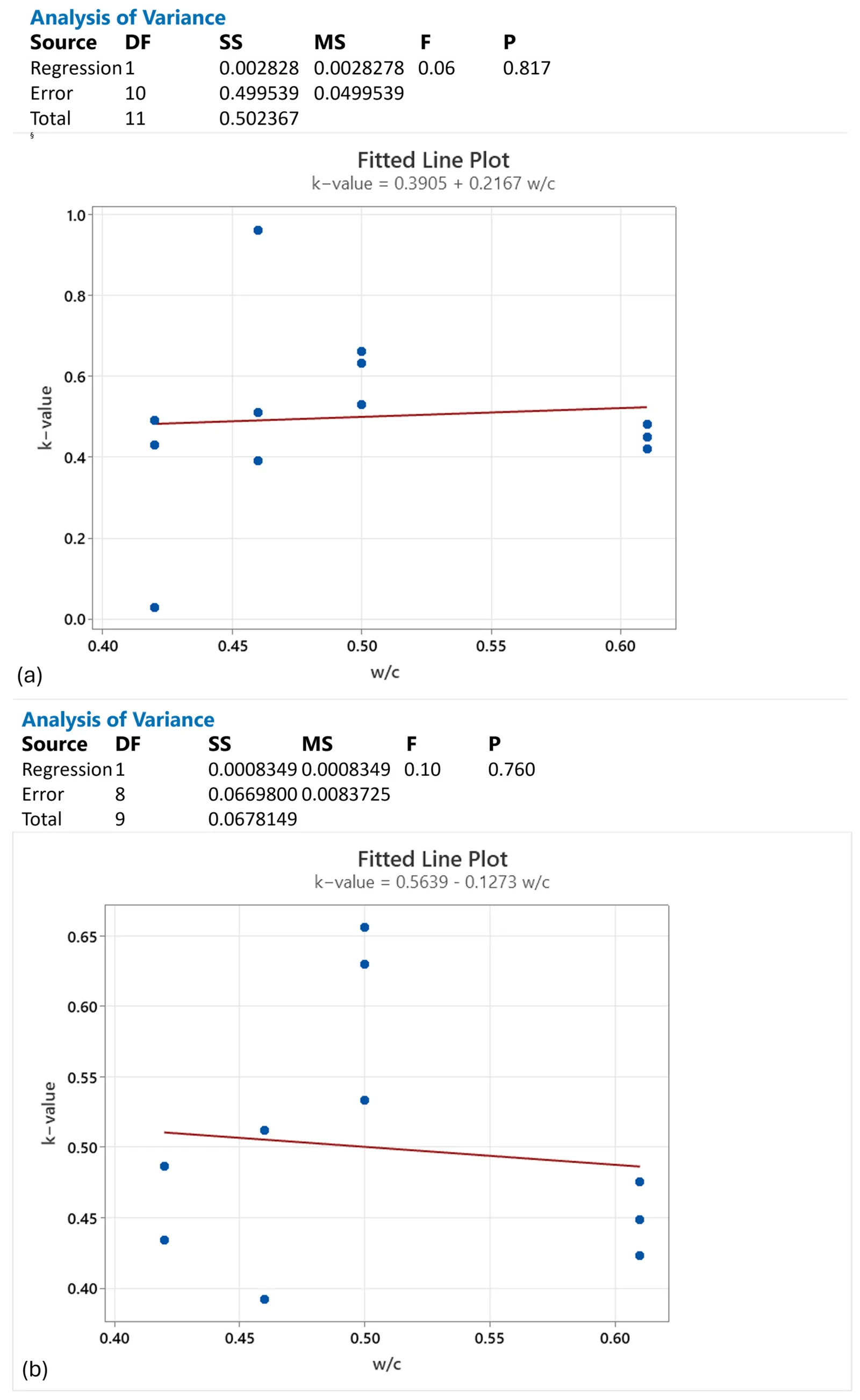
(**a**) k-value vs. w/c for all the original data. (**b**) k-value vs. w/c after removing the outliers.

A sensitivity analysis was performed on the relationship between k-value and w/c. The results showed the absence of a statistically significant relationship, irrespective of the outlier data points in the experimental data. After performing the sensitivity analysis, it was observed that the removal of outliers did not significantly alter the R-squared and *p*-values (Figure 5), which further confirm that no significant relationship exists across all experimental datasets. Table 8 shows the strength values with outliers removed. Therefore, the strength results in Table 8 are slightly different from Table 7.

**Figure 6:**
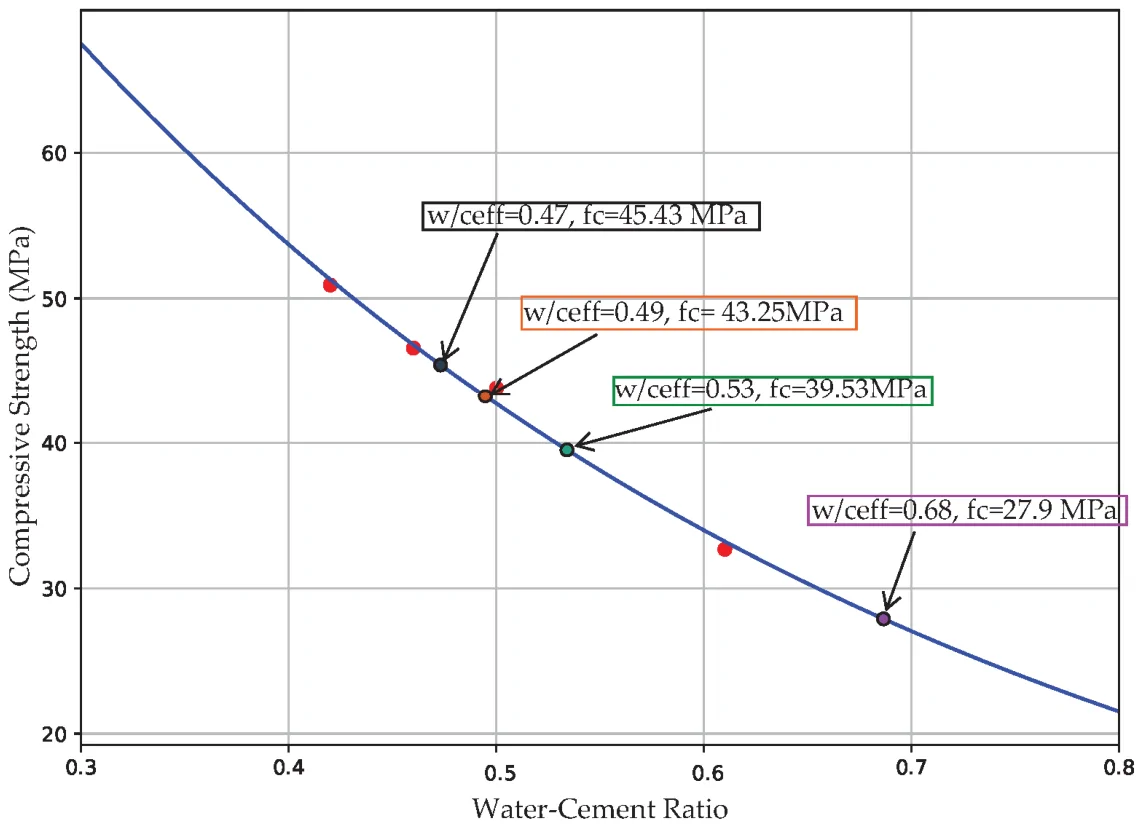
Abram’s equation fit of reference concrete strength in addition to concrete with 80% CEMII and 20% GP compressive strength plot at each water–binder ratio, based on computation on Python 3.13.18 (Google Colab; Google LLC, Mountain View, CA, USA).

The relation between compressive strength and w/c for the reference concrete mix was established through Abram’s curve, calculated in Equation (1). The compressive strength results of GP concrete were plotted on this curve to determine the corresponding w/c. To estimate both coefficients, Python, which is a programming language, was used for more accurate and faster estimation, code is provided in Supplementary Materials Python code 2. The water–cement ratio and compressive strength of reference concrete (CEM II) were used as inputs to the code. Figure 6 shows the compressive strength of GP concrete plotted on the reference concrete strength curve, and Table 8 presents the compressive strength of tested samples having 80% CEM II and 20% GP in addition to their effective water–binder ratios, which can be calculated using Equation (1). The effective water–binder ratios were higher than the actual water–binder ratio because concrete containing GP exhibits lower compressive strength than the corresponding reference concrete.

### 3.4. Glass Powder k-Value

Table 9 shows the calculation of the k-value for glass powder as an SCM in concrete using Equation (3), where Ceqv is estimated from the effective water–binder ratio using Equation (2), and A represents the amount of glass powder quantity used in the mix. For each water–binder ratio, a different k-value of glass was obtained. An overall k-value was determined by averaging the values obtained at the different water–binder ratios, resulting in a k-value of 0.5.

### 3.5. Empirical Performance Design Efficiency Factor k_SAI_

#### 3.5.1. Glass Powder k_SAI_

**Figure 7:**
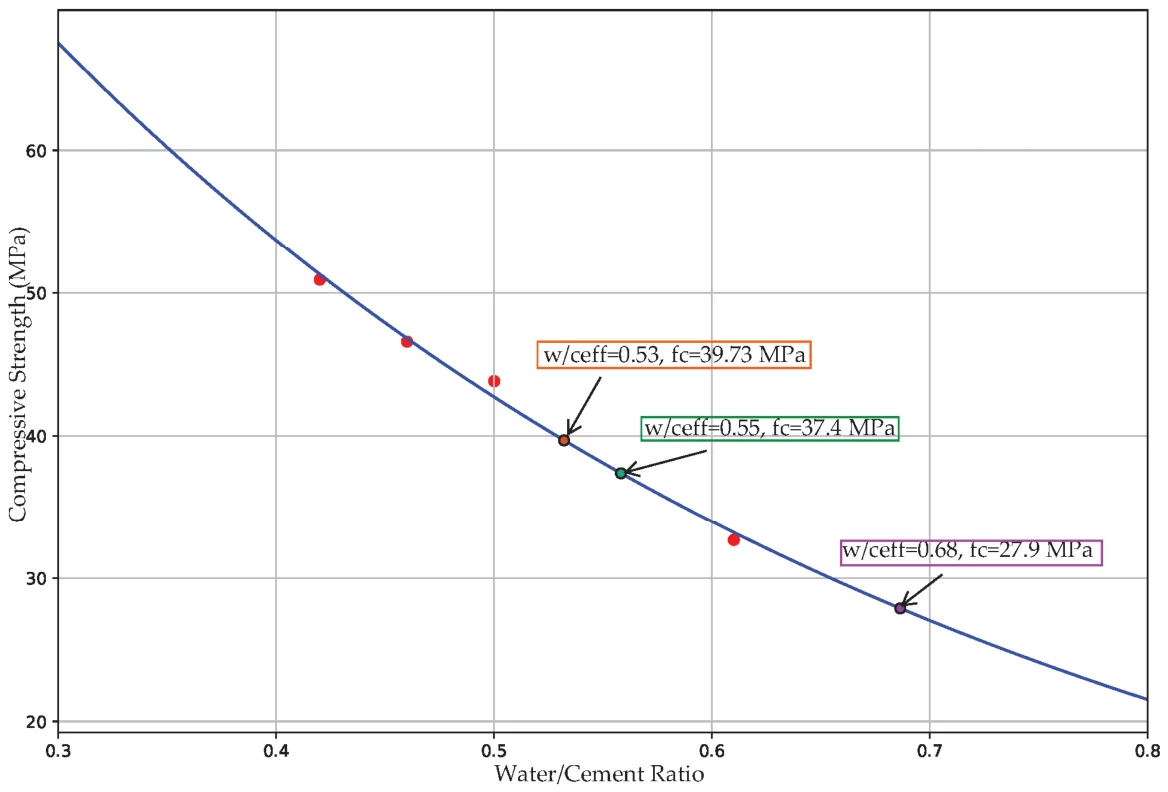
Abram’s equation curve of reference concrete CEM II, with SAI based concrete strength of 20% glass powder concrete, based on computation in Python.

Figure 7 represents Abram’s curve fitted to the reference concrete data, together with effective w/c and compressive strength derived using the SAI approach. This follows the same approach used for conventional k-value evaluation, where the k-value of GP is derived from the Abram’s curve for reference concrete, thereby allowing the determination of k_SAI_. The SAI was combined with the reference concrete compressive strength at different w/c ratios to derive the corresponding values. Table 10 presents the individual k_SAI_ values, as well as the mean k_SAI_ of 0.43. This indicates that glass powder provides approximately 42% of the strength contribution of cement. The evaluation was performed using Python 3.13.18 (Google Colab), which is a programming language, for quick and more accurate results, code is provided in Supplementary Materials Python code 3.

The result of k_SAI_ was 16% less than that of the k-value. This suggests that k_SAI_ can be used as a preliminary assessment method for SCMs, as its value is within the range of the conventional k-value. However, k_SAI_ tends to produce lower values than the conventional k-value, which may lead to higher estimated SCM replacement requirements. This limitation is particularly relevant for SCMs with high alkali content, where the method may provide an overly conservative assessment and should, therefore, be interpreted with caution. This calculated k_SAI_ is limited to the tested glass type, with tested oxide composition, and particle size distribution, which influences the reactivity of the investigated glass.

#### 3.5.2. Screening k-Value with k_SAI_

The k_SAI_ method is intended as a rapid screening tool for estimating the k-value of glass powder, or other SCMs, at a given replacement level. The literature has shown that the k-value depends on the replacement percentage of the SCM, particularly for glass powder [30]. For a glass powder replacement level of 20%, the proposed method yielded a k_SAI_ value of 0.43, while the corresponding experimentally determined k-value was 0.5. This shows that the k_SAI_ provides an estimation of k-value, which can be obtained with minimal experimental effort for a certain replacement percentage of glass powder and potentially other SCMs. However, the proposed method is not intended to replace the conventional k-value determination procedure. Instead, it offers a practical preliminary assessment, particularly when evaluating different glass powders with varying chemical compositions or particle size distributions. It should be noted that these k-value results, in proximity with k_SAI_, are possible for SAI tested by ASTM C1866, which uses the same SCM replacement as that used in the concrete mixture. In contrast, the activity index determined by the EN standard is based on a fixed SCM replacement level of 25% replacement.

## 4. Equivalent Performance of Concrete with Glass Powder

To validate the k-value, the compressive strength of a concrete mixture containing GP is compared with that of reference concrete. Two concrete recipes, one containing only GP as an SCM and the other containing GP as SCM, and recycled aggregates are evaluated for equivalent performance. The quantity of GP doubled when a k-value of 0.5 was applied, resulting in an increase in binder content from 370 kg/m^3^ to 444 kg/m^3^. Therefore, the GP replacement level increased to approximately 33%.

### 4.1. Compressive Strength

SIS-CEN/TR 16639:2014 [21] specifies that concrete with an SCM fulfils the requirements of the X0, XF1, XF3 exposure classes, if its compressive strength is comparable to that of the reference concrete or that meets the fixed specified limit values of 20 and 33 MPa at 7 and 28 days, respectively, at a w/c of 0.55. In this study, since glass powder is not a standardized SCM, concrete containing GP with a k-value of 0.5 was compared with the reference concrete at w/c 0.5 to meet the requirement of the XF3 exposure class of maximum 0.5 w/c. It was observed that compressive strength increased by 4% compared with the reference. This indicates that GP concrete with a 0.5 k-value satisfies the compressive strength requirements of ECPC, referring to Table 11.

### 4.2. Circular Concrete Equivalence

**Figure 8:**
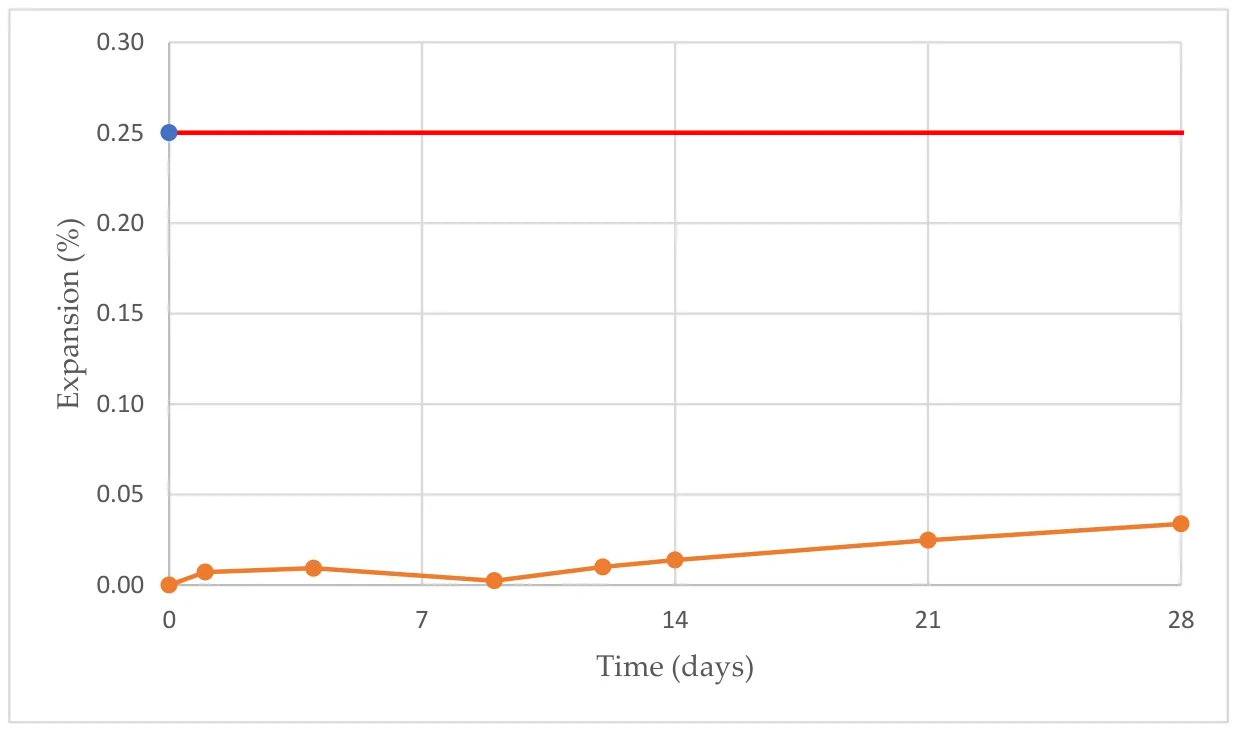
ASR expansion results for recycled aggregate CCA.

In an upscaling activity related to project RE:glass, recycled aggregates (CCAs) were tested to assess the possibility of replacing natural aggregates [31]. The results show that coarse CCA achieved a frost resistance classification of category F1, and the concrete exhibited very good frost resistance (refer to Table 12). In addition, the measured ASR expansions were consistently low across all samples (Figure 8). At 14 days, the average expansion across all the samples was below the limit and remained so at 28 days.

The results indicate that the tested coarse CCA meets the frost resistance requirements for the XF1 and XF3 exposure classes.

**Figure 9:**
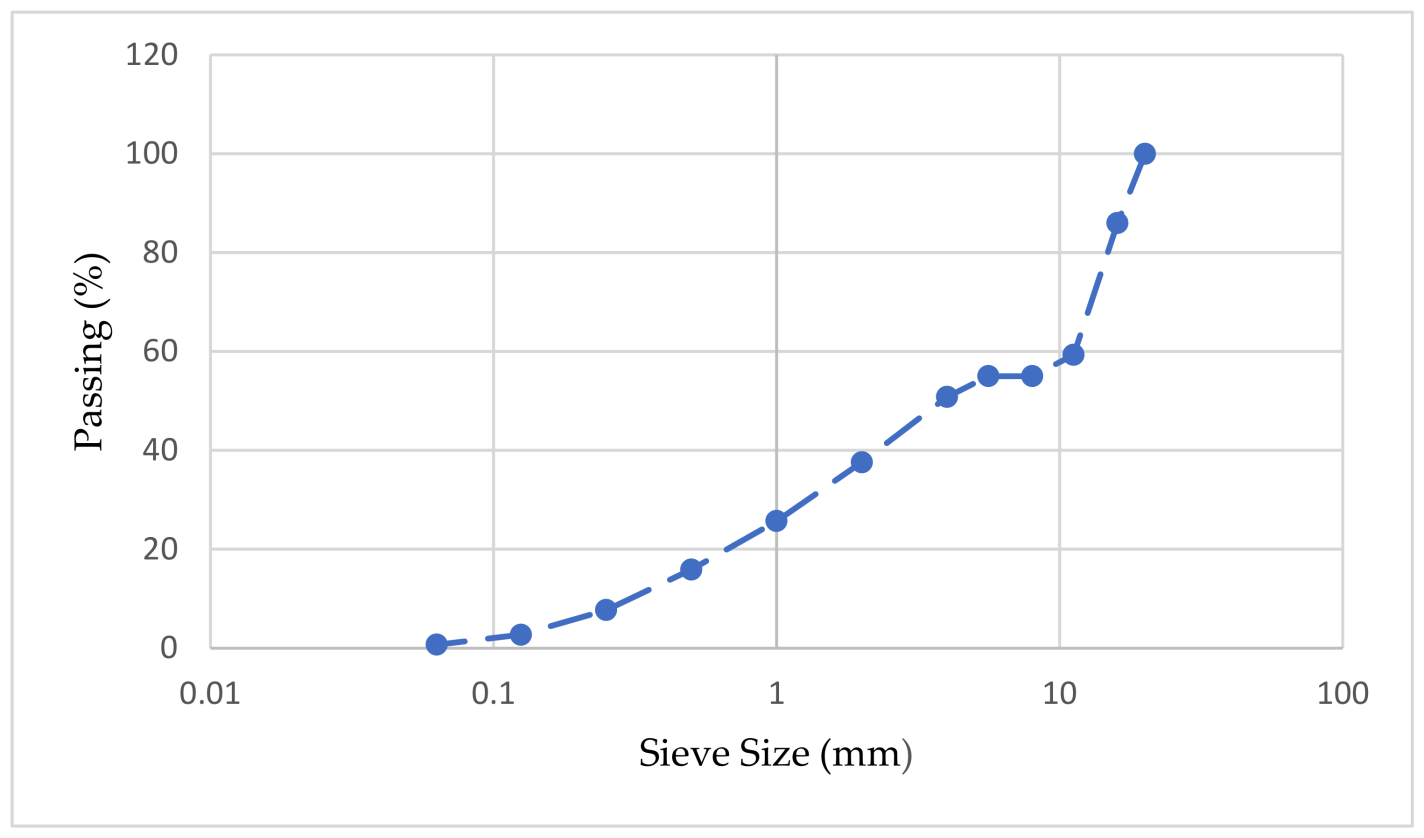
Particle size distribution of recycled aggregate CCA 0–4 and 8–16 fractions combined.

Three cylinders of size 200 × 100 mm were prepared for the evaluation of strength equivalence between concrete containing 100% CCA aggregates (fine and coarse) and reference concrete. CCA concrete incorporated 20% GP with a k-value of 0.5, reaching a replacement level of 33%. As CCA fines are present in a 0/4 mm fraction, there is a new reference concrete (REF 0/4) containing 0/4 crushed concrete fines and CCA kGP, in addition to the corresponding compressive strength. Table 13 provides the REF 0/4 and CCA kGP recipe mix, in addition to the corresponding compressive strengths, indicating a 7% increase for CCA kGP concrete. This demonstrates the equivalency of CCA kGP concrete to REF 0/4, as both frost-resistant aggregates and concrete, and the compressive strength requirements are satisfied. Table 14 and Figure 9 provide the properties of the recycled CCA aggregates. The CCA used in this study consists of recycled aggregates produced from concrete leftovers.

## 5. Conclusions

This study demonstrates the feasibility of using a mechanistic–empirical framework for the rapid evaluation of SCMs with minimal material consumption. Mechanically activated glass powder, evaluated within a water–binder range of 0.4–0.6, satisfied pozzolanic requirements when 69% of the particles were smaller than 20 micrometers. SAI was used to identify the best replacement level, and 20% replacement was found to provide the best performance.

The concrete containing 20% GP exhibited lower compressive strength (39 MPa) than the reference (43 MPa). The k-value of GP was derived using Abram’s law from the compressive strengths of reference and GP concrete at water–binder ratios of 0.42, 0.46, 0.5 and 0.61. This results in a k-value of 0.5, which is shown to have so significant relationship with water–binder.

When the glass quantity in the mixes was adjusted using a k-value of 0.5, the concrete achieved a compressive strength of 45 MPa, which is comparable to the reference (43 MPa), with an increase of approximately 4%.

The SAI-based efficiency factor was derived by combining the SAI with the reference compressive strength to estimate the compressive strength of glass powder mortar. This works as a strength prediction tool, with k_SAI_ = 0.43 close to the investigated k-value of 0.5. This indicates that the SAI approach can serve as a rapid method for screening SCM efficiency and k-value performance on the strength of cementitious materials.

Concrete produced with 100% recycled aggregates and GP adjusted using a k-value of 0.5 achieved comparable strength to that of the reference concrete, demonstrating that both recycled aggregates and GP can serve as sustainable alternatives. Furthermore, concrete incorporating k-value GP and recycled aggregate CCA reaches comparable strength and satisfies the requirements of the ECPC framework. Therefore, it can be considered suitable for frost resistance application in exposure classes XF1 and XF3, confirming its potential for durable and sustainable concrete application.

## Figures and Tables

**Table 1:** ASR vs. GP as an SCM.

Glass Alkali Content	Method Used	Glass Size	ASR Mitigation	Ref.
Na_2_O 11% K_2_O 0.56%	ABNT NBR 15577	Passing 75 microns	At 50% replacement	[18]
Na_2_O 13.78% K_2_O 0.66%	ASTM C1567	Passing 53 microns	At 20% replacement	[16]
Na_2_O 13.2% K_2_O 0.12%	ASTM C311	Passing 75 microns	At 20% replacement (reduces the expansion in comparison to reference)	[5]
Na_2_O 10.27% K_2_O 1.27%	JGJ52-2006	Passing 75 microns	At 20% replacement	[17]

**Table 2:** Oxide content of soda glass GP and CEMII [3].

Oxide Content (%)			
	GP	CEM II	Combined 80% CEMII 20% GP
SiO_2_	71.2	20.6	30.7
Al_2_O_3_	0.36	5.6	4.5
Fe_2_O_3_	0.44	3	2.5
CaO	9.33	62.4	51.7
MgO	3.86	3	3.17
Na_2_O	13.2	1–2	3.4–4.2
K_2_O	0.04	1–2	0.8–1.6
SO_3_	<0.1	4	<3.22
Density (kg/m^3^)	2500	3100–3200	2980–3060

**Table 3:** Concrete mix proportions, cement replacement with 20% GP of different w/c, 61, 50, 46, and 42 refers to 0.61, 0.5, 0.46, and 0.42 w/c, respectively, in addition to k-value and k_SAI_.

Concrete Mix (kg/m^3^)										
Sample	CEM II61	GP 61	CEM II50	GP 50	CEM II46	GP 46	CEM II42	GP42	kGP	k_SAI_GP
Cement (kg)	370	296	370	296	370	296	370	296	296	296
GP (kg)	-	74	-	74	-	74	-	74	148	110
Water (L)	230	230	185	185	170	170	155	155	185	185
Sand (kg)	954	932	1004	1001	1026	1014	1049	1044	900	952
Coarse (kg)	758	749	806	804	824	814	842	839	804	804

**Table 4:** Mortar recipes for SAI according to ASTM C1866.

SAI Recipe (ASTM C1866)				
	CEM II	20CGP	25CGP	30CGP
Cement (kg)	500	400	375	350
Glass (kg)	-	100	125	150
Sand (kg)	1375	1375	1375	1375
Water (L)	242	242	242	242

**Table 5:** 7-day SAI of different GP replacements.

		7 Days fc (MPa)		
	CEM II	20CGP	25CGP	30CGP
	30.1	27.9	22.3	20.7
	25.8	23.70	22.8	23.1
	29.6	22.4	26.2	26
Mean (MPa)	28.5	24.7	23.8	23.2
SAI (%)		86	83	82
fc, StdDev	2.35	2.87	2.12	2.65

**Table 6:** SAI of mortar cubes of 80% OPC and 20% GP at 28 days of curing age.

28 Days fc (MPa)		
	CEM II	20CGP
	38.51	34.42
	36.65	26.84
	32.11	30.21
Mean	35.76	30.49
StdDev	2.68	3.79
SAI %		85

**Table 7:** Workability and compressive strength of concrete with CEM II/A-LL and CEM II/A-LL with 20% GP at different w/c.

Concrete Properties								
Samples	CEM II 61	GP 61	CEM II 50	GP 50	CEM II 46	GP 46	CEM II 42	GP 42
Slump (mm)	190	200	180	185	185	190	180	220
**fc, mean (MPa)**	**32.6**	**27.9**	**43.8**	**39.53**	**46.5**	**44.3**	**50.9**	**45.4**
fc, StdDev	3	1	2.93	1.4	3.96	2.85	5.14	2.35
GP/CEMII (%)	85		88		95		89	

**Table 8:** Compressive strength and effective water–binder ratios of 20% GP concrete.

Sample	w/b	20% GP Concrete Compressive Strength (MPa)	Plotted Effective w/c
GP 61	0.61	27.9	0.68
GP 50	0.5	39.53	0.54
GP 46	0.46	43.25	0.48
GP 42	0.42	45.43	0.47

**Table 9:** Glass powder k-value calculation.

k-Value Calculation				
w/b	Binders (kg)	Effective w/c	Ceqv (kg)	k-Value
0.61	370	0.68	329.2	0.45
0.5	370	0.53	340.7	0.61
0.46	370	0.49	341.6	0.62
0.42	370	0.47	319.2	0.31
Mean k-value				0.5

**Table 10:** Mortar-based (SAI) k_SAI_ determination based on computation in Python.

GP SAI-Based Efficiency Coefficient						
	Water (L)	Cement (kg)	Glass (kg)	w/c Effective	fc (MPa)	k_SAI_
20CGP—0.46	169	296	74	0.53	39.73	0.31
20CGP—0.5	182	296	74	0.55	37.4	0.47
20CGP—0.61	226	296	74	0.68	27.9	0.49
Mean k_SAI_						0.43

**Table 11:** Properties of reference concrete vs. concrete incorporating k-value glass powder.

	k-Value Concrete Properties	Reference Concrete
	kGP	CEM II 50
Workability (mm)	195	180
Compressive strength (MPa)	45.5	43.8
StDev	4.5	2.93
Water/Cement	-	0.5
Water/Binder	0.42	-

**Table 12:** Coarse CCA aggregates and concrete with 100% coarse CCA frost resistance testing.

Frost Resistance of CCA 8/16 Aggregates and Concrete with 100% 8/16 CCA				
Aggregates	Fragmentation (%)	Category	Concrete	Category
CCA	1%	F1	100% 8/16 CCA	Very good

**Table 13:** Recipe mix and compressive strength of CCA kGP and REF 0/4 concrete.

Recipe Mix and Compressive Strength of CCA kGP Concrete		
	REF 0/4	CCA kGP
Cement (kg/m^3^)	370	296
GP (kg/m^3^)	-	148
0/4 Crushed rocks	728	-
8/16 Crushed rocks	865	-
0/4 CCA (kg/m^3^)	-	656
8/16 CCA (kg/m^3^)	-	854
Water (L/m^3^)	185	185
Compressive strength (MPa)	31.5	33.8
fc, StDev	1.4	1.7
Water/cement	0.5	-
Water/Binder	-	0.42

**Table 14:** CCA recycled aggregate properties.

Aggregate Fraction (mm)	Water Absorption (%)	Density (kg/m^3^)
0/4	5.7	2550
8/16	2.5	2640

## Data Availability

The original contributions presented in this study are included in the article/Supplementary Material. Further inquiries can be directed to the corresponding author.

## Supplementary Materials

The following supporting information can be downloaded at: https://www.mdpi.com/article/10.3390/ma19214740/s1, Python codes used in this article, Python code 1: Code for drawing compressive strength vs w/c graph estimating K1 and K2 coefficient factors; Python code 2: Code for drawing compressive strength vs w/c ratio graph and estimating K1 and K2 coefficient factors, in addition to plotting the results for the glass powder results on the curve (alternative for code 1); Python code 3: Code for finding keff.
