# Supplementary material for: Mechanistic Empirical Design for Circular Concrete Performance with Glass Powder
Source: Materials (Basel). 2026 Nov 8;19(21):4740. doi: 10.3390/ma19214740 (PMC13765317; doi:10.3390/ma19214740)
Supplement: Supplementary file 1 [file materials-19-04740-s001.zip › materials-4576788-supplementary.pdf]

1- Code for drawing compressive strength vs w/c ratio graph and estimating K1 and K2 coefficient factors

```
import numpy as np

import matplotlib.pyplot as plt

from scipy.optimize import curve_fit

# === Step 1: Input your data ===

w_c_ratios = np.array([0.42, 0.46, 0.50, 0.61])  # Water-to-cement ratios

compressive_strengths = np.array([50.92, 46.59, 43.83, 32.69])  # Corresponding strengths in MPa

# === Step 2: Define Abram's equation ===

def abraham_equation(w_c, k1, k2):

    return k1 / (k2 ** w_c)

# === Step 3: Fit the model to your data ===

initial_guess = [100, 2]

params, _ = curve_fit(abraham_equation, w_c_ratios, compressive_strengths, p0=initial_guess)

k1_fit, k2_fit = params

# === Step 4: Calculate y-axis intersection (w/c = 0) ===

w_c_zero = 0

y_intercept = abraham_equation(w_c_zero, k1_fit, k2_fit)

# === Step 5: Plot the results ===

w_c_fit = np.linspace(0, max(w_c_ratios), 100)

fc_fit = abraham_equation(w_c_fit, k1_fit, k2_fit)

plt.figure(figsize=(8, 5))

plt.plot(w_c_fit, fc_fit, color='blue', label=f'Fitted Curve\nk1={k1_fit:.2f}, k2={k2_fit:.2f}')

plt.scatter(w_c_zero, y_intercept, color='green', label=f'K1\n({w_c_zero}, {y_intercept:.2f})')

plt.xlabel("Water/Cement Ratio (w/c)")

plt.ylabel("Compressive Strength (MPa)")

plt.grid(True)

plt.tight_layout()

plt.show()

# === Step 6: Output the fitted parameters ===

print(f"Estimated k1: {k1_fit:.2f} MPa")
```

```
print(f"Estimated k2: {k2_fit:.2f} (unitless)")
```

2- Code for drawing compressive strength vs w/c ratio graph and estimating K1 and K2 coefficient factors, in addition to plotting the results for the glass powder results on the curve (alternative for code 1)

```
import numpy as np

import matplotlib.pyplot as plt

from scipy.optimize import curve_fit

# === Step 1: Input your data ===

w_c_ratios = np.array([0.42, 0.46, 0.50, 0.61])  # Water-to-cement ratios

compressive_strengths = np.array([50.92, 46.59, 43.83, 32.69])  # Corresponding strengths in MPa

# === Step 2: Define Abram's equation ===

def abraham_equation(w_c, k1, k2):

    return k1 / (k2 ** w_c)

# === Step 3: Fit the model to your data ===

initial_guess = [100, 2]

params, _ = curve_fit(abraham_equation, w_c_ratios, compressive_strengths, p0=initial_guess)

k1_fit, k2_fit = params

# === Step 4: Calculate y-axis intersection (w/c = 0) ===

w_c_zero = 0.0

y_intercept = abraham_equation(w_c_zero, k1_fit, k2_fit)

# === Step 5: Prepare domain and fitted curve ===

x_min, x_max = 0.3, max(w_c_ratios)  # you can extend x_max if needed (e.g., 0.8 or 1.0)

w_c_fit = np.linspace(x_min, x_max, 400)

fc_fit = abraham_equation(w_c_fit, k1_fit, k2_fit)

# === NEW: Define your horizontal lines (y-only) ===

y_lines = [27.9, 38.77, 44.28, 45.44]  # your given y-values (MPa)

colors = ['#8e44ad', '#16a085', '#d35400', '#2c3e50']  # distinct colors

# Analytic intersection: y = k1 / (k2**x) -> x = ln(k1/y) / ln(k2)

def intersection_x(y, k1, k2):

    # Guard against invalid values
```

```python
    if y <= 0:
        return np.nan
    if k2 <= 0 or np.isclose(k2, 1.0):
        # k2<=0 invalid for log; k2==1 makes the curve constant and intersection undefined
        return np.nan
    x = np.log(k1 / y) / np.log(k2)
    return x
# Compute intersections
intersection_points = []
for y in y_lines:
    x_int = intersection_x(y, k1_fit, k2_fit)
    intersection_points.append((y, x_int))
# === Plot ===
plt.figure(figsize=(10, 7))
plt.scatter(w_c_ratios, compressive_strengths, color='red', label='Data')
plt.plot(w_c_fit, fc_fit, color='blue')
# Draw horizontal lines and mark intersections
for (y, x_int), c in zip(intersection_points, colors):
    # Mark intersection if finite
    if np.isfinite(x_int):
        # Mark only if inside the plotted x-range; else you can still annotate outside
        in_range = (x_min <= x_int <= x_max)
        if in_range:
            plt.scatter(x_int, y, color=c, edgecolor='black', zorder=5)
            plt.annotate(f"w/c = {x_int:.3f}",
                         (x_int, y),
                         textcoords="offset points", xytext=(6, 6),
                         ha='left', color=c, fontsize=9)
        else:
            # Optional: draw a small marker at the edge to indicate it's outside
            # Comment out if you prefer not to mark
```

```python
            edge_x = x_max if x_int > x_max else x_min
            plt.scatter(edge_x, y, color=c, marker='x', zorder=5)
            plt.annotate(f"w/c = {x_int:.3f} (outside range)",
                         (edge_x, y),
                         textcoords="offset points", xytext=(6, 6),
                         ha='left', color=c, fontsize=8)
plt.title("Abram's Equation Fit to Concrete Strength")
plt.xlabel("Water/Cement Ratio (w/c)")
plt.ylabel("Compressive Strength (MPa)")
plt.xlim(x_min, x_max)
plt.grid(True)
plt.tight_layout()
plt.show()
# === Step 6: Print fitted parameters and intersections ===
print(f"Estimated k1: {k1_fit:.4f} MPa")
print(f"Estimated k2: {k2_fit:.4f} (unitless)")
print(f"Y-axis intersection (w/c = 0): {y_intercept:.4f} MPa\n")
print("Intersections of horizontal lines with fitted curve:")
for y, x in intersection_points:
    if np.isfinite(x):
        domain_note = "" if (x_min <= x <= x_max) else " (outside plotted x-range)"
        print(f"  y = {y:.2f} MPa -> w/c = {x:.5f}{domain_note}")
    else:
      print(f"  y = {y:.2f} MPa -> no valid intersection (check k2 and y)")
```

3- Code for finding k-eff, can be used also to find k-value

```python
# ====================================================================
# Imports (MISSING in your code)
```

```
#
==============================================================
====
import pandas as pd
import numpy as np
# --- simple helper functions (MISSING in your code) ---
def t_ci_mean(x, alpha=0.05):
    x = np.asarray(x)
    x = x[~np.isnan(x)]
    n = len(x)
    if n < 2:
        return np.mean(x), np.nan, np.nan
    mean = np.mean(x)
    se = np.std(x, ddof=1) / np.sqrt(n)
    tcrit = 2.0  # simplified (small sample anyway)
    return mean, mean - tcrit * se, mean + tcrit * se
def bootstrap_ci_mean(x, alpha=0.05, B=20000, random_state=42):
    rng = np.random.default_rng(random_state)
    x = np.asarray(x)
    x = x[~np.isnan(x)]
    means = rng.choice(x, size=(B, len(x)), replace=True).mean(axis=1)
    lo = np.percentile(means, 100 * alpha / 2)
    hi = np.percentile(means, 100 * (1 - alpha / 2))
    return means.mean(), lo, hi
def normality_tests(x):
    return {"note": "Skipped (SciPy not included)"}
# --- global parameters
alpha = 0.05
expected_k = 0.0
# ====================================================
# DATA
# ====================================================
```

```
data = [
    ("20CGP-0.46", 0.46, 43.18, 0.495, 169, 296, 74),
    ("20CGP-0.50", 0.50, 40.63, 0.522, 182, 296, 74),
    ("20CGP-0.61", 0.61, 30.3, 0.65, 226, 296, 74),
]
df = pd.DataFrame(data, columns=[
    "mix_id",
    "w_b",
    "fc_MPa",
    "X_ekv",
    "water_w_kgperm3",
    "cement_c_kgperm3",
    "glass_powder_a_kgperm3",
])
# ============================================================
# k_eff CALCULATION
# ============================================================
W = df["water_w_kgperm3"].to_numpy(dtype=float)
C = df["cement_c_kgperm3"].to_numpy(dtype=float)
A = df["glass_powder_a_kgperm3"].to_numpy(dtype=float)
X = df["X_ekv"].to_numpy(dtype=float)
keff = (W / X - C) / A
df["k_i"] = keff
# ============================================================
# PRINT RESULTS
# ============================================================
print("\n=== Per-mix k_eff ===")
for _, row in df.iterrows():
    print(
        f"{row['mix_id']}: k_eff = {row['k_i']:.4f}  "
        f"(W={row['water_w_kgperm3']}, C={row['cement_c_kgperm3']}, "
```

```
        f"glass={row['glass_powder_a_kgperm3']}, X_ekv={row['X_ekv']:.3f}, "
        f"fc={row['fc_MPa']:.2f})"
    )
# ============================================================
# SUMMARY
# ============================================================
n = len(keff)
k_mean, ci_lo, ci_hi = t_ci_mean(keff, alpha)
k_med = float(np.nanmedian(keff))
k_iqr = float(np.nanpercentile(keff, 75) - np.nanpercentile(keff, 25))
k_min, k_max = float(np.nanmin(keff)), float(np.nanmax(keff))
b_mean, b_lo, b_hi = bootstrap_ci_mean(keff, alpha)
print("\n=== Summary on keff ===")
print(f"n = {n}")
print(f"mean = {k_mean:.4f}")
print(f"median = {k_med:.4f}")
print(f"IQR = {k_iqr:.4f}")
print(f"min = {k_min:.4f}, max = {k_max:.4f}")
```
